# The Story of the Insane from Year to Year

**Published:** 1889-07-27

**Authors:** 


### The Story of the Insane from Year to Year.

Asylum Reports.

### LANCASHIRE COUNTY ASYLUM, RAINHILL.

In our summary of the reports for 1887 we stated it was impossible to draw any reliable conclusion from the statistics of any one year, as the conditions were not alike in any two asylums, and that even the same asylum differed greatly from year to year. The report of the Rainhill Asylum for 1888 bears out our statement. It began the year with 850 patients, and ended with 1,200. There were 539 admissions, 118 discharges, and 116 deaths. The recoveries stand at 16·88 per cent. on the admissions, but if "transfers" be omitted the percentage would rise to 19·73, while the average for 38 years was 33·25. Among the admissions were 71 paralytics, 54 epileptics, 19 idiots, and 112 were chronic maniacs. The superintendent of one of the neighbouring asylums thinks he could cure 60 per cent. of the admissions if he had a hospital specially arranged for acute cases. Doubtless Dr. Wigglesworth would gladly hand him these over for a beginning in the new system which is to do so much for us. The death-rate was 11·83. One of the deaths was from typhoid fever. In 65 cases the insanity was caused by drink, and in 51 it was hereditary. Dr. Rogers retired on his pension, and the visitors say that "the recent resignation of Dr. Rogers entails upon the committee the loss of a most able, experienced, and zealous superintendent," a recognition of his worth with which everyone acquainted with him will cordially agree. Dr. Wigglesworth, one of the assistant medical officers, reigns in his stead.

### CAMBRIDGE ASYLUM.

This asylum now issues its thirty-first annual report, and it has increased its numbers from 115 to 393 during the thirty-one years of its existence. Ninety-eight patients were admitted last year, 66 were discharged, and 31 died. The recoveries stand at 35·7 per cent. on the admissions, and the deaths at 7·9 on the average number resident. There is a deficiency of room in the asylum, but additions for 80 patients are being made, and in the meantime upwards of 50 cases are boarded out at the Northampton County Asylum. The Cambridge Asylum must be one of the very few in England where the attendants do not wear uniform. The visitors close their last report to Quarter Sessions, as follows: "Your committee cannot close their report without special reference to Dr. E. C. Rogers, who, by the ability and courtsey with which he has acted as medical superintendent for the period of five years and upwards, has won their full confidence, and to Dr. E. J. G. Crallan, who for a rather longer term has discharged the duties of assistant medical officer to their complete satisfaction." The medical superintendent's report is a very short one, but perhaps it contains all that is necessary for such a document to contain. Certainly, purely medical details are out of place, and we notice they are becoming rarer and rarer as the years go by. The weekly rate is 10s. 9¾d.

### ST. LUKE'S HOSPITAL, LONDON.

We learn from the report of this hospital that 45 patients were admitted during 1888, 59 were discharged, and 6 died. The percentage of recoveries seems to be calculated, not on the total number of cases admitted, but on the curable cases only, and it comes out at 52·27. There does not seem to be any good reason for forsaking the usual course in this matter. The deaths were 2·3 per cent., calculated also in an unusual way—namely, on the total number under treatment. As statistics are chiefly valuable for comparison, it seems a pity that the usual course is not followed at St. Luke's. The expenditure during the year was over £12,000, and it is a pleasing feature in the report that the income exceeded the expenditure.

### IPSWICH BOROUGH ASYLUM.

This hospital was opened in 1870, and on the last day of that year it contained 108 patients. At the end of 1888 the numbers had risen to 207, but 16 were private patients, and many of these were chargeable to outlying districts, so that the borough is to be congratulated on not having any alarming increase of insanity. Eighty-six cases were admitted during the year, 76 were discharged, and 29 died. The recoveries stand at 37·2 per cent. on the admissions, and the deaths at 12·5 per cent on the average number resident. Eleven of the deaths were due to phthisis, which seems a very high mortality from this disease for so small an asylum. Dr. Chevallier has the following remarks on idiots: "Idiots and imbeciles of a tender age, most unsuitable for admixture with either acute or chronic cases of ordinary insanity, continue to be admitted. At an idiot asylum they would be instructed to a considerable extent; but here there are no means of developing any faculties that are capable of cultivation and improvement, and are altogether out of place. I fear there is not much prospect of legislation for their benefit for a long time to come." If compulsory legislation be meant undoubtedly Dr. Chevallier is right, but authorities have already the power of building for idiots and imbeciles; or the committee of visitors might add a separate ward for them. This has been done at one county asylum, and it is said to be perfectly successful. The weekly rate is nearly 10s. 8d.

